# Cowpea speed breeding using regulated growth chamber conditions and seeds of oven-dried immature pods potentially accommodates eight generations per year

**DOI:** 10.1186/s13007-022-00938-3

**Published:** 2022-08-29

**Authors:** Offiong Ukpong Edet, Takayoshi Ishii

**Affiliations:** 1 Arid Land Research Center, Tottori University, Tottori, Japan; 2 Department of Crop Science, University of Calabar, Calabar, Nigeria

**Keywords:** Cowpea, Light-emitting diode chamber, Metal halide lamp chamber, Accelerated generation advance, CO_2_ supplementation, Cultivation of immature seeds, Improved hand pollination

## Abstract

**Background:**

Cowpea is a dryland crop with potential to improve food security in sub-Saharan Africa, where it is mostly produced and consumed. Contemporary plant improvement technologies, including genome editing, marker-assisted selection, and optimized transformation protocols, are being deployed to improve cowpea characteristics. Integrating speed breeding with these technologies would accelerate genetic gain in cowpea breeding. There are established speed breeding protocols for other important legumes, such as soybean, peanut, and chickpea, but none has been previously reported for cowpea.

**Results:**

With the aid of regulated growth conditions in two different chamber types, as well as the cultivation of new plant generations from seeds of oven-dried immature pods, we developed and validated, for the first time, an efficient speed breeding protocol that accommodates approximately seven to eight breeding generations per year for 3 cowpea genotypes. The 3 cowpea genotypes were evaluated under controlled growth conditions in light-emitting diode and metal halide lamp chambers to determine the effect of CO_2_ supplementation on flowering and maturation durations, optimum conditions for plant growth, cross pollination, and pod development. Elevated CO_2_ concentration had no influence on either flowering time or pod development. Adequate temperature, relative humidity and light intensity improved plant development and the rate of successful hand pollination, and  cultivating seeds of 11-day-old immature pods oven-dried at 39 °C for 2 days resulted in at least a 62% reduction in the time between pollination and sowing of the next plant generation. The plants cultivated from seeds of the oven-dried immature pods showed no defect at any stage of development.

**Conclusions:**

Using the speed breeding protocol developed in this study, cowpea breeding cycles can be increased from the traditional one cycle per year in the field to as many as 8 generations per year in regulated growth chamber conditions. This protocol has no special technical requirements; hence, it can be implemented in any standard growth chamber. This would fast-track development, testing, validation, and utilization of improved cowpea cultivars.

**Supplementary Information:**

The online version contains supplementary material available at 10.1186/s13007-022-00938-3.

## Background

Cowpea (*Vigna unguiculata* [L.] Walp.) is an important diploid (2n = 22), autogamous legume species of African origin, which is suitable for dryland farming. Cowpea is widely consumed as a source of protein and utilized as a fodder or pasture crop for livestock in sub-Saharan Africa. Its abilities to check weeds and biologically fix nitrogen through symbiosis with compatible nitrogen-fixing rhizobia are advantageous for crop rotation and intercropping systems [1–3]. In 2019, Africa produced 96.8% of the 8.9 million tonnes of global cowpea dry seeds, and Nigeria, the world’s largest producer and consumer of cowpea, accounted for about 40% of global production [4]. Notwithstanding its position as the highest producer of cowpea, Nigeria still imports about 0.5 million tonne of cowpea annually to meet its domestic needs [5]. To address this production/consumption deficit in Nigeria and other African countries where millions of households consume cowpea [6], high throughput phenotyping technologies and genomic resources are being deployed to develop high yielding and stress resilient varieties for various agro-ecologies [7–11]. Technical developments in cowpea research have resulted in an improved transformation method [12], genome and transcriptome sequence data [13, 14], and the discovery of two functional variants of centromere-specific histone 3 (CENH3) with unequal contributions to centromeric functions [15]. The identification and characterization of these cowpea CENH3 variants has provided new opportunities and challenges for further beneficial manipulation of the cowpea genome, in particular, the potential of centromere-mediated genome elimination to induce haploidy [16–18].

Cowpea is an annual seasonal crop, whose cultivation is restricted to the warm months of the year. As such, only one breeding generation is feasible in a year, which is inadequate for breeding programs that require many breeding generations. Molecular breeding techniques, such as marker-assisted selection and genome editing, are used to fast-track the development and validation of improved cultivars, but they are currently limited by the seasonal life cycle of cowpea. Speed breeding, a technique which circumvents the limitations imposed by natural field conditions and allows the life cycle of a crop to be shortened, is a promising approach to increase the number of cowpea breeding generations per year. Speed breeding protocols have been developed for important crops [19–22], including some legumes [23–25], but none has previously been reported for cowpea. In many speed breeding protocols, the key development is a method to substantially reduce the time from sowing to flowering of the crop. Regulation of photoperiod and temperature, light quality modification, and CO_2_ supplementation have successfully reduced days to flowering of some crops [19, 26, 27]. Because some cowpea genotypes, including the three we studied, are photo-insensitive [28, 29], we thought a speed breeding protocol involving the manipulation of photoperiod would not be useful in the breeding of photo-insensitive cowpea genotypes. In addition, a recent report on the optimum conditions for cowpea cultivation in growth chambers shows that temperatures outside the range we adopted in our study negatively impact different stages of cowpea development and reproduction [30]. Although increase in temperature can reduce the length of cowpea life cycle [31], exposing cowpea to high temperatures results in flower abortion and significant reduction in pod formation [30]. Cultivation of air-dried immature seeds greatly reduced the reproductive duration of soybean [25]. Therefore, we investigated the sensitivity of three cowpea genotypes to CO_2_ supplementation, determined appropriate chamber conditions for hand pollination, and tested and confirmed the value of cultivating cowpea from immature seeds oven-dried at an optimum temperature and duration in increasing the number of cowpea breeding generations per year.

This study presents, for the first time, a proven simple and effective speed breeding protocol that can potentially accommodate approximately seven to eight breeding generations per year for three cultivars of cowpea (Sasaque, IT86D-1010 and IT97K-499-35). We used different growth chamber conditions at different growth stages to achieve enhanced vegetative growth, improved hand pollination and rapid pod development. Cultivation of seeds from oven-dried immature pods led to an appreciable increase in the number of breeding generations achievable per year.

## Results

### Regulated growth conditions improve the success rate of cowpea hand pollination

Successful hand pollination of cowpea is largely dependent on the environmental conditions under which the crop is grown. The success rates of crosses recorded under various growth conditions in the light-emitting diode (LED) and metal halide lamp chambers indicate that low to moderate temperature, high relative humidity, and moderate light intensity facilitate improved hand pollination (Table 1, Additional file 1: Table S1). From a total of 36 crosses per treatment, 9 in each replicate, successful hand pollinations in the LED chambers ranged from 60 to 80% per replicate in LED (−) and 65–70% per replicate in LED (+) (Additional file 1: Table S1). There was no significant difference in the success of the crosses made in LED (−) and LED (+) (p > 0.05), indicating that difference in CO_2_ concentration ([CO_2_]) has no effect on cowpea pollination. Hand pollinations were significantly more successful in the LED chambers than the metal halide lamp chambers, especially in metal halide lamp A and B chambers (p < 0.001), where light intensity was more than three-fold higher than the light intensity in the LED chambers (Table 1, Additional file 1: Table S1). Average success frequency of hand pollinations in metal halide lamp C was significantly higher than the average success rates in metal halide lamp chambers A and B (p < 0.001) (Additional file 1: Table S1), which indicates that hand pollination of cowpea benefits from an adequate combination of light intensity, temperature and relative humidity. Based on our results, we recommend 230–420 µmol m^−2^ s^−1^ light intensity, 70/80% (day/night) relative humidity and 22–23/25–29 °C (night/day) temperature as optimum conditions for successful hand pollination of cowpea. Under the growth conditions we adopted for hand pollination in our chambers (Table 1), we achieved 65–70% average success in hand pollination in the LED chambers and 50% average success in metal halide chamber C within the first week of flowering. This is critical, as slow crosses can extend the duration of crop cycles, thereby reducing the number of breeding generations possible per year.

**Table 1:** Description of growth chambers and plant cultivation conditions. Photoperiod was 10 h light in all chambers

Chamber Description	LED (−)	LED (+)	Metal halide A (−)	Metal halide B (−)	Metal halide C (−)
Internal dimensions (H × L × B) cm	107 × 63 × 53	107 × 63 × 53	198 × 258 × 166	198 × 258 × 166	198 × 258 × 166
Light source and intensity (µmolm^−2^ s^−1^)	White LED; 230	White LED; 230	Metal halide; 700	Metal halide; 700	Metal halide; 420
Temperature (^o^C, day/night)	29/23	29/23	29/23	29/23	25/22
Relative humidity for germination and plant growth (%, day/night)	60/70	60/70	60/70	60/70	60/70
Relative humidity for hand pollination (%, day/night)	70/80	70/80	70/80	60/70	70/80

*LED,* light-emitting diode; (−), no CO_2_ supplementation; (+), CO_2_ supplemented. Two each of LED (−) and LED (+) chambers were used in the study

### CO_2_ supplementation has no effect on flowering and maturity of cowpea

**Fig. 1:**
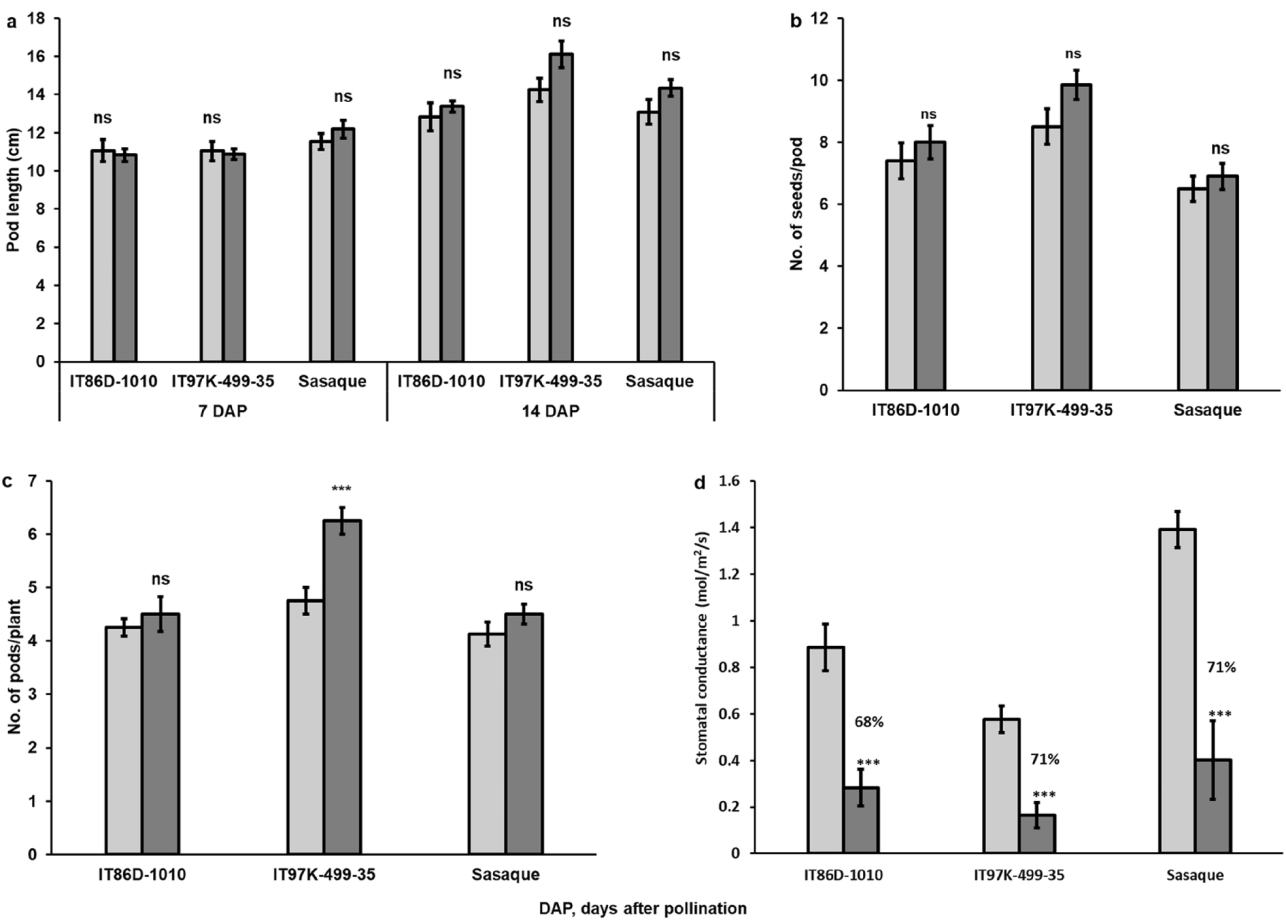
Effect of CO_2_ supplementation on pod yield components and stomatal conductance. Effect of CO_2_ supplementation effect on **a** pod length (n = 20), **b** number of seeds/pod (n = 20), **c** number of pods/plant (n = 8) and **d** stomatal conductance (n = 8). ns, no significant difference; ***significantly different (p < 0.001)

Neither time to flowering nor time to maturity of the three genotypes was affected by CO_2_ supplementation; there was also no observable difference in the germination of seeds harvested from the field or growth chambers with or without CO_2_ supplementation. To determine pod elongation duration, we measured pod lengths of the same pods at 7, 11, and 14 days after pollination (DAP). Pod lengths at 14 DAP were the same as the values measured at 11 DAP, hence we have reported here pod lengths measured at 7 and 14 DAP (Fig. 1a). Pod lengths and numbers of seeds per pod of the 3 genotypes were not significantly affected by CO_2_ supplementation (Fig. 1a, b). Number of pods per plant increased significantly under CO_2_ supplementation in only IT97K-499-35 (Fig. 1c). However, the elevated [CO_2_] resulted in almost equal reduction of stomatal conductance (g_sw_) in the 3 genotypes (Fig. 1d). Because CO_2_ supplementation significantly increased the number of pods formed in IT97K-499-35, but not in Sasaque and IT86D-1010, it is likely that genetic variation influences cowpea’s sensitivity to CO_2_ supplementation, reduced stomatal conductance or both.

### Cultivation of cowpea from oven-dried seeds increases the potential number of breeding generations per year

**Fig. 2:**
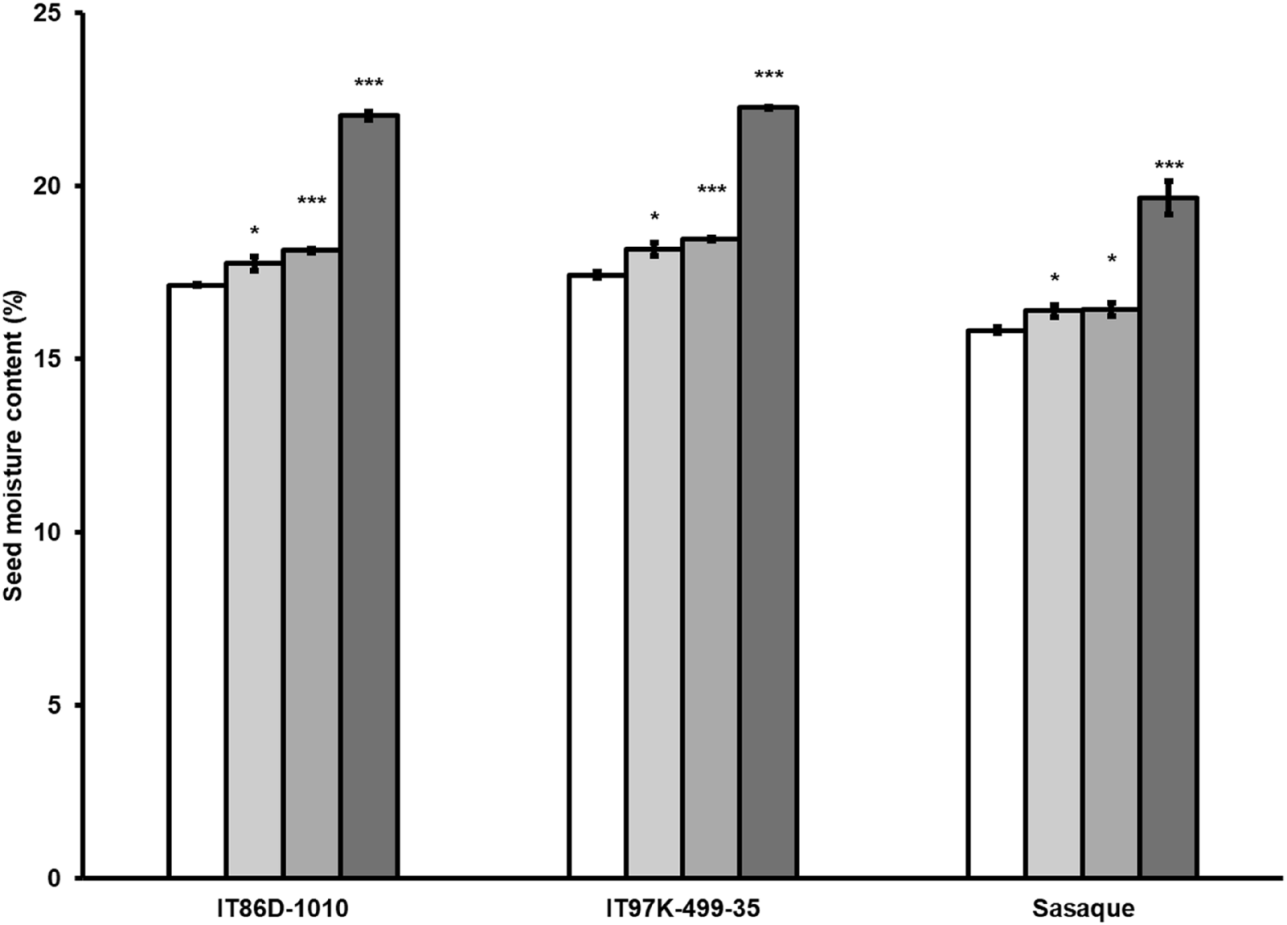
Comparison of moisture contents of seeds allowed to dry on plants (white bar) with 14-day-old seeds oven-dried at 25 °C for 4 days (brighter grey bar), seeds of 11-day-old pods oven-dried at 39 °C for 2 days with silica gel included in the drying oven (bright grey bar), and seeds of 11-day-old pods oven-dried at 39 °C for 2 days without silica gel included in the drying oven (dark grey bar). * and ***significantly higher at p ≤ 0.05 and 0.001, respectively

We cultivated seeds of oven-dried immature pods to shorten the reproductive phase of cowpea. As cowpea attains maximum pod and seed development by 15 days after anthesis [32], in the F_1_ generation, we harvested immature pods at 14 DAP and oven-dried at 25 °C for 4 days before sowing the seeds to test viability and measure emergence rate of seedlings in comparison with emergence rate of seedlings from seeds allowed to naturally dry on plants before harvesting (Table 2). Emergence rates and heights of seedlings from the 14-day-old oven-dried seeds and seeds allowed to naturally dry on plants did not significantly differ (Table 2). To further reduce the length of the reproductive phase of cowpea, in F_2_ generation, we studied the germinability of seeds of oven-dried pods from 7 to 11 DAP. Each harvested pod was oven-dried for 2 days at 25, 30, 35, 36, 37, 38, 39, 40, 41, 42, 43, 44 or 45 °C before seeds were sown. At 7 days after sowing (DAS), no seedling emerged from all the seeds of pods oven-dried at temperatures higher than 42 °C for 2 days, seeds of 9-day-old pods oven-dried for 2 days at temperatures lower than 38 °C, seeds of 7- and 8-day-old pods oven-dried for 2 days at all the temperatures studied. The emergence rate of seedlings from seeds of 11-day-old  pods  oven-dried for 2 days at 39 °C was not significantly different from the emergence rate of seedlings from the control (seeds allowed to dry on plant before harvesting); hence, more 11-day-old pods were dried at 39 °C for 1 day, 1 day with addition of silica gel and 2 days with addition of silica gel (Table 2). Silica gel was included in the drying oven as a desiccant to quicken the drying process. In all the genotypes except the 11-day-old 97 K × 86D F_2_ hybrid (oven-dried at 39 °C for 2 days without silica gel), there was no significant difference in emergence rates between the control, 14-day-old pods oven-dried for 4 days at 25 °C, 11-day-old pods oven-dried for 2 days at 39 °C with or without silica gel (Table 2). Heights of seedlings at 10 DAS were also not significantly different between the control, seeds of 14-day-old pods  oven-dried at 25 °C for 4 days, seeds of 11-day-old pods oven-dried at 39 °C for 2 days with silica gel in the drying oven for all the genotypes, and 11-day-old F_2_ hybrid seeds oven-dried for 2 days at 39 °C without silica gel in the drying oven for all the F_2_ hybrids. Although heights of seedlings of the parental genotypes cultivated from seeds allowed to naturally dry and the seedlings from the seeds of 11-day-old pods oven-dried at 39 °C for 2 days without silica gel significantly differed, there was no significant difference in heights of seedlings from the seeds of 11-day-old pods oven-dried at 39 °C for 2 days with or without silica gel (p > 0.05). Days to flowering between the control plants and the plants cultivated with the seeds of 11-day-old pods oven-dried at 39 °C for 2 days without silica gel did not significantly differ (p > 0.05) (Table 3), hence the plants cultivated from the seeds of 11-day-old pods oven-dried at 39 °C for 2 days with silica gel included in the drying oven were not maintained after flowering. However, to obtain vigorous seedlings, we recommend the inclusion of silica gel while oven-drying 11-day-old pods at 39 °C for 2 days before sowing. Including silica gel in the oven-drying of the 11-day-old pods at 39 °C for 2 days significantly reduced the seed moisture content as compared to the moisture content of seeds of 11-day-old pods oven-dried at the same temperature and duration without silica gel (p < 0.001) (Fig. 2). The inclusion of silica gel in the drying process brought the moisture content of the seeds of 11-day-old pods closer to the moisture content of the seeds allowed to dry on plant (Fig. 2), which is likely to account for the observed improved vigor of the seedlings (Table 2).

**Table 2:** Comparison of emergence rate and height of seedlings from mature dry seeds and immature oven-dried seeds of three cowpea genotypes and their hybrids

Pod age at harvesting (DAP)	Oven-drying		Average seedling emergence rate 7 DAS ± SEM (%)						Seedling height 10 DAS ± SEM (cm)					
	Temperature (^o^C)	Duration (day)	IT86D-1010	IT97K-499-35	Sasaque	SAS × 86D	97 K × 86D	SAS × 97 K	IT86D-1010	IT97K-499-35	Sasaque	SAS × 86D	SAS × 97 K	97 K × 86D
Control	**N/A**	**N/A**	**87 ± 7**	**83 ± 3**	**93 ± 3**	**87 ± 7**	**87 ± 3**	**90 ± 6**	**11.0 ± 0.3**	**11.0 ± 0.3**	**9.9 ± 0.1**	**10.0 ± 0.3**	**10.2 ± 0.4**	**9.3 ± 0.4**
14	**25**	**4**	**83 ± 3**	**87 ± 7**	**90 ± 6**	**90 ± 6**	**83 ± 3**	**93 ± 3**	**10.6 ± 0.4**	**10.8 ± 0.4**	**9.5 ± 0.4**	**9.6 ± 0.3**	**10.0 ± 0.3**	**10.1 ± 0.3**
11	25	2	17 ± 3***	13 ± 3***	20 ± 6***	20 ± 6**	20 ± 6***	13 ± 3***	4.9 ± 0.6***	4.7 ± 0.4***	4.2 ± 0.4***	4.2 ± 0.44***	5.2 ± 0.54***	4.4 ± 0.34***
11	30	2	17 ± 3***	17 ± 3***	23 ± 3***	17 ± 7**	17 ± 3***	20 ± 6***	5.2 ± 0.6***	4.4 ± 0.3***	4.2 ± 0.3***	4.2 ± 0.34***	4.0 ± 0.34***	5.6 ± 0.24***
11	35	2	20 ± 6**	17 ± 3***	27 ± 3***	20 ± 6**	17 ± 3***	23 ± 3***	5.4 ± 0.2***	5.3 ± 0.4***	5.0 ± 0.4***	5.0 ± 0.44***	5.1 ± 0.34***	5.6 ± 0.24***
11	36	2	23 ± 3***	17 ± 3***	27 ± 3***	20 ± 6**	17 ± 3***	20 ± 6***	5.2 ± 0.6***	4.6 ± 0.4***	5.0 ± 0.3***	4.9 ± 0.34***	4.6 ± 0.44***	5.3 ± 0.54***
11	37	2	33 ± 3**	33 ± 3***	37 ± 3***	30 ± 6**	33 ± 3***	37 ± 3***	5.0 ± 0.3***	4.9 ± 0.2***	5.2 ± 0.2***	5.3 ± 0.24***	4.6 ± 0.24***	5.2 ± 0.24***
11	38	2	33 ± 3**	27 ± 3***	40 ± 6**	37 ± 3**	30 ± 6***	37 ± 3***	6.0 ± 0.2***	6.0 ± 0.4***	5.8 ± 0.2***	6.0 ± 0.34***	5.8 ± 0.44***	6.0 ± 0.34***
11	**39**	**2**	**73 ± 3**	**70 ± 6**	**77 ± 3**	**70 ± 6**	67 ± 3*	**70 ± 6**	9.8 ± 0.3*	9.9 ± 0.2*	9.0 ± 0.2*	**9.0 ± 0.3**	**10.2 ± 0.1**	**9.5 ± 0.3**
11	40	2	60 ± 6*	57 ± 3**	67 ± 3*	63 ± 3*	50 ± 6**	63 ± 3*	7.0 ± 0.3***	6.4 ± 0.2***	5.8 ± 0.4***	5.6 ± 0.24***	6.6 ± 0.24***	7.0 ± 0.34**
11	41	2	57 ± 3*	50 ± 6**	57 ± 3**	53 ± 3*	43 ± 3***	53 ± 3*	6.4 ± 0.2***	5.7 ± 0.4***	5.8 ± 0.4***	6.0 ± 0.34***	5.9 ± 0.54***	6.0 ± 0.34***
11	42	2	17 ± 3***	13 ± 3***	20 ± 6***	17 ± 3***	17 ± 7***	23 ± 3***	3.8 ± 0.2***	3.3 ± 0.3***	3.9 ± 0.3***	3.5 ± 0.24***	3.8 ± 0.24***	4.0 ± 0.34***
10	25	2	13 ± 3***	13 ± 3***	13 ± 3***	17 ± 3***	17 ± 3***	13 ± 3***	3.3 ± 0.3***	3.7 ± 0.3***	3.7 ± 0.3***	3.6 ± 0.24***	3.6 ± 0.24***	3.7 ± 0.34***
10	30	2	17 ± 3***	20 ± 6***	17 ± 6***	17 ± 3***	17 ± 3***	20 ± 6***	3.4 ± 0.2***	3.3 ± 0.3***	3.8 ± 0.2***	3.8 ± 0.24***	3.3 ± 0.34***	3.4 ± 0.24***
10	35	2	17 ± 3***	13 ± 3***	23 ± 3***	20 ± 6**	13 ± 3***	23 ± 3***	3.8 ± 0.2 ***	3.4 ± 0.3***	3.6 ± 0.2***	3.6 ± 0.24***	3.4 ± 0.44***	3.8 ± 0.24***
10	36	2	23 ± 3***	17 ± 3***	23 ± 3***	27 ± 3***	20 ± 6***	27 ± 3***	4.4 ± 0.4***	4.0 ± 0.4***	4.6 ± 0.2***	4.2 ± 0.44***	4.0 ± 0.44***	4.8 ± 0.24***
10	37	2	23 ± 3***	17 ± 3***	27 ± 3***	30 ± 6**	20 ± 6***	30 ± 6**	4.4 ± 0.2***	4.5 ± 0.2***	3.8 ± 0.4***	4.0 ± 0.44***	4.5 ± 0.24***	4.2 ± 0.24***
10	38	2	27 ± 3***	23 ± 7***	27 ± 3***	27 ± 7***	23 ± 3***	30 ± 6**	5.0 ± 0.4***	4.4 ± 0.2***	4.2 ± 0.2***	4.2 ± 0.24***	4.6 ± 0.44***	4.8 ± 0.44***
10	39	2	53 ± 3**	47 ± 3***	53 ± 3**	57 ± 3*	50 ± 6**	53 ± 3**	5.2 ± 0.4***	5.0 ± 0.3***	5.2 ± 0.2***	5.2 ± 0.24***	5.0 ± 0.34***	5.2 ± 0.44***
10	40	2	43 ± 3**	37 ± 3***	47 ± 3**	47 ± 3**	37 ± 3***	43 ± 3**	4.6 ± 0.2***	4.4 ± 0.2***	4.6 ± 0.2***	4.6 ± 0.24***	4.6 ± 0.24***	4.4 ± 0.24***
10	41	2	47 ± 3**	33 ± 3***	47 ± 3**	43 ± 3**	37 ± 7**	43 ± 3**	4.8 ± 0.2***	4.4 ± 0.2***	4.4 ± 0.2***	4.2 ± 0.24***	4.6 ± 0.24***	4.8 ± 0.24***
10	42	2	13 ± 3***	13 ± 3***	17 ± 3***	17 ± 3***	13 ± 3***	17 ± 3***	2.8 ± 0.1***	2.9 ± 0.1***	3.2 ± 0.2***	3.2 ± 0.24***	2.9 ± 0.14***	3.1 ± 0.24***
9	38	2	17 ± 3***	13 ± 3***	20 ± 10**	17 ± 3***	17 ± 3***	23 ± 3 ***	3.4 ± 0.2***	3.3 ± 0.3***	3.6 ± 0.2***	3.6 ± 0.24***	3.6 ± 0.24***	3.2 ± 0.24***
9	39	2	23 ± 3***	20 ± 6***	23 ± 3***	27 ± 3***	20 ± 6***	27 ± 3***	3.8 ± 0.2***	3.4 ± 0.2***	3.6 ± 0.2***	3.4 ± 0.24***	3.6 ± 0.24***	3.8 ± 0.24***
9	40	2	17 ± 3***	13 ± 3***	20 ± 6***	13 ± 3***	17 ± 3***	23 ± 3***	3.8 ± 0.2***	3.3 ± 0.3***	3.4 ± 0.2***	3.7 ± 0.34***	3.4 ± 0.24***	3.8 ± 0.24***
9	41	2	20 ± 6**	13 ± 3***	20 ± 6***	20 ± 6**	17 ± 3***	20 ± 10**	3.1 ± 0.1***	3.7 ± 0.3***	3.4 ± 0.2***	3.4 ± 0.24***	3.6 ± 0.24***	3.1 ± 0.14***
11	39	1	37 ± 3**	33 ± 3***	40 ± 6**	33 ± 3**	37 ± 3***	37 ± 7**	5.2 ± 0.2***	5.0 ± 0.3***	4.2 ± 0.2***	4.4 ± 0.24***	5.0 ± 0.34***	5.0 ± 0.34***
11	39 + silica gel	1	37 ± 3**	30 ± 6***	43 ± 3**	33 ± 3**	30 ± 6***	37 ± 7**	5.6 ± 0.2***	5.6 ± 0.4***	5.0 ± 0.3***	5.4 ± 0.24***	5.6 ± 0.44***	5.2 ± 0.44***
11	**39 + silica gel**	**2**	**83 ± 3**	**77 ± 3**	**87 ± 3**	**80 ± 6**	**80 ± 6**	**83 ± 3**	**10.5 ± 0.4**	**10.3 ± 0.4**	**9.6 ± 0.4**	**9.9 ± 0.4**	**10.3 ± 0.4**	**10.2 ± 0.5**

Bold text, emphasis on optimum oven-drying conditions; *, **, ***, significantly lower than control at p ≤ 0.05, 0.01, 0.001, respectively; The germination rate and corresponding SEM values are approximated to the nearest whole numbers. To measure emergence rates of seedlings under the experimental conditions, 10 seeds were sown in three replications (30 seeds/treatment) and emergence rates were recorded at 7 DAS. T-tests were applied to compare each of the mean values of emergence rates and heights of seedlings cultivated from the different categories of oven-dried seeds and seedlings cultivated from mature seeds allowed to dry on plants before harvesting. At 7 DAS, no seedling emerged from all the seeds oven-dried at temperatures higher than 42 °C, seeds of 9-day-old pods oven-dried for 2 days at temperatures lower than 38 °C, seeds of 7- and 8-day-old pods oven-dried for 2 days at all the temperatures reported here. Hybrid seeds for oven-drying were harvested 14 DAP in F_1_, and 7–11 DAP in F_2_

DAP, days after pollination; DAS, days after sowing; N/A, not applicable; Control**,** pods allowed to dry on plant before harvesting; SAS, Sasaque; 86D, IT86D-1010; 97 K, IT97K-499-35; SEM, standard error of the mean

**Table 3:** Increased number of breeding generations per year facilitated by regulated growth conditions and cultivation of plants from seeds of oven-dried immature pods

Genotype	Seed source	Growth chamber type	Days to 50% flowering ± SD (n = 3)	Pod age (DAP) at harvest	Duration of pod oven drying (days (^o^C))	Days from sowing to the next generation	Potential No. of cycles/year
Parents (preliminary trial)							
Sasaque	Metal halide (−)	LED (+)	32 ± 1.5	29	N/A	61	6.0
Sasaque	Metal halide (−)	LED (−)	32 ± 1.5	29	N/A	61	6.0
IT86D-1010	Metal halide (−)	LED (+)	40 ± 1.0	32	N/A	72	5.1
IT86D-1010	Metal halide (−)	LED (−)	40 ± 1.0	32	N/A	72	5.1
IT97K-499-35	Field	LED (+)	44 ± 1.0	31	N/A	75	4.9
IT97K-499-35	Field	LED (−)	44 ± 1.0	31	N/A	75	4.9
Parents (main trial)							
Sasaque	LED (+)	LED (+)	32 ± 1.5	14	4 (25)	50	7.3
Sasaque	LED (−)	LED (−)	32 ± 1.5	14	4 (25)	50	7.3
IT86D-1010	LED (+)	LED (+)	40 ± 1.0	14	4 (25)	58	6.3
IT86D-1010	LED (−)	LED (−)	40 ± 1.0	14	4 (25)	58	6.3
IT97K-499-35	LED (+)	LED (+)	44 ± 1.0	14	4 (25)	62	5.9
IT97K-499-35	LED (−)	LED (−)	44 ± 1.0	14	4 (25)	62	5.9
F_1_ generation							
Sasaque	LED (−)	Metal halide A	32 ± 1.5	11	2 (39)	45	8.1
IT86D-1010	LED (−)	Metal halide A	40 ± 1.0	11	2 (39)	53	6.8
IT97K-499-35	LED (−)	Metal halide A	44 ± 1.0	11	2 (39)	57	6.4
Sasaque × IT86D-1010	LED (−)	Metal halide A	35 ± 1.5	11	2 (39)	48	7.6
Sasaque × IT97K-499-35	LED (−)	Metal halide A	35 ± 1.7	11	2 (39)	48	7.6
IT97K-499-35 × IT86D-1010	LED (−)	Metal halide A	42 ± 2.1	11	2 (39)	55	6.6
F_2_ generation							
Sasaque	Metal halide A	Metal halide A	32 ± 1.5	11	2 (39)	45	8.1
IT86D-1010	Metal halide A	Metal halide A	40 ± 1.0	11	2 (39)	53	6.8
IT97K-499-35	Metal halide A	Metal halide A	44 ± 1.0	11	2 (39)	57	6.4
Sasaque × IT86D-1010	Metal halide A	Metal halide A	35 ± 1.5	11	2 (39)	48	7.6
Sasaque × IT97K-499-35	Metal halide A	Metal halide A	35 ± 1.7	11	2 (39)	48	7.6
IT97K-499-35 × IT86D-1010	Metal halide A	Metal halide A	42 ± 2.1	11	2 (39)	55	6.6

To determine average days to flowering, each generation of the main study was considered a replicate for the parents, whereas the F_1_ and F_2_ plants were cultivated in three replicates, four plants in each replicate (n = 3)

DAP, days after pollination; LED, light-emitting diodes; (−), no CO_2_ supplementation; (+), CO_2_ supplemented; N/A, not applicable; SD, standard deviation

**Fig. 3:**
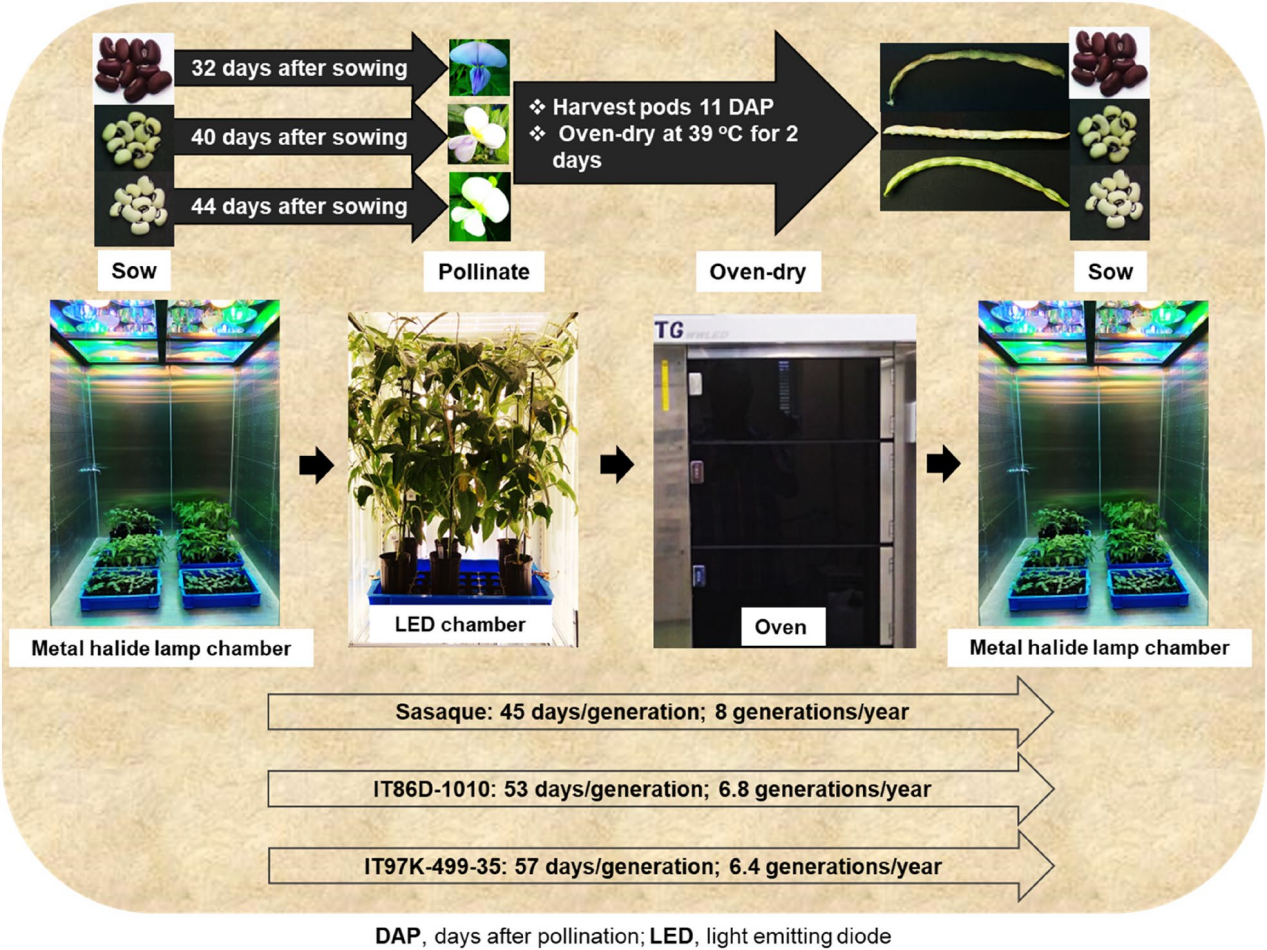
Shortened life cycles of 3 cowpea genotypes facilitated by sowing of oven-dried 11-day-old seeds

Emergence rates and heights of seedlings in treatments other than 14-day-old pods oven-dried for 4 days and 11-day-old pods oven-dried at 39 °C for 2 days with or without silica gel were significantly lower than control (Table 2). From our results, to attain the maximum number of breeding generations of cowpea per year, the most appropriate time for immature pods to be harvested is 11 DAP, and to achieve a satisfactory emergence rate of seedlings, the 11-day-old pods should be oven-dried for 2 days at 39 °C with or without silica gel. Under our growth chamber conditions, Sasaque, IT86D-1010 and IT97K-499-35 require average of 61, 72 and 75 days to complete one crop cycle (drying of at least 50% of the pods on each plant), resulting in 6, 5.1 and 4.9 potential breeding generations per year, respectively (Table 3); this is a valuable increase from the one generation per year that is currently realized under field conditions. Integrating cultivation of seeds of oven-dried immature pods increased the number of possible cycles per year (Table 3, Fig. 3). By sowing seeds from 11-day-old pods oven-dried for 2 days at 39 °C, the duration of one breeding cycle was 45 days for Sasaque, 53 days for IT86D-1010 and 57 days for IT97K-499-35, resulting in a gain of approximately two additional breeding generations for each of the genotypes (Table 3, Fig. 3). As compared to cultivating seeds naturally allowed to dry on plant before harvesting, cultivating 11-day-old seeds, reduced the time between pollination and sowing of the next generation by 18 days (62%) for Sasaque, 21 days (65%) for IT86D-1010, and 20 days (65%) for IT97K-499-35 (Table 3). Plants germinated and grown in metal halide lamp chambers A and B grew faster and to larger size, and flowered 2 to 3 days earlier than the plants cultivated in the LED chambers. Consequently, to achieve the maximum number of generations per year, we recommend that plants should be raised under similar growth conditions as the settings in the metal halide chamber A until flower buds initiate, at which stage the plants should be transferred to similar growth conditions as the settings in the LED chambers for cross pollination and development of pods (Fig. 3).

**Fig. 4:**
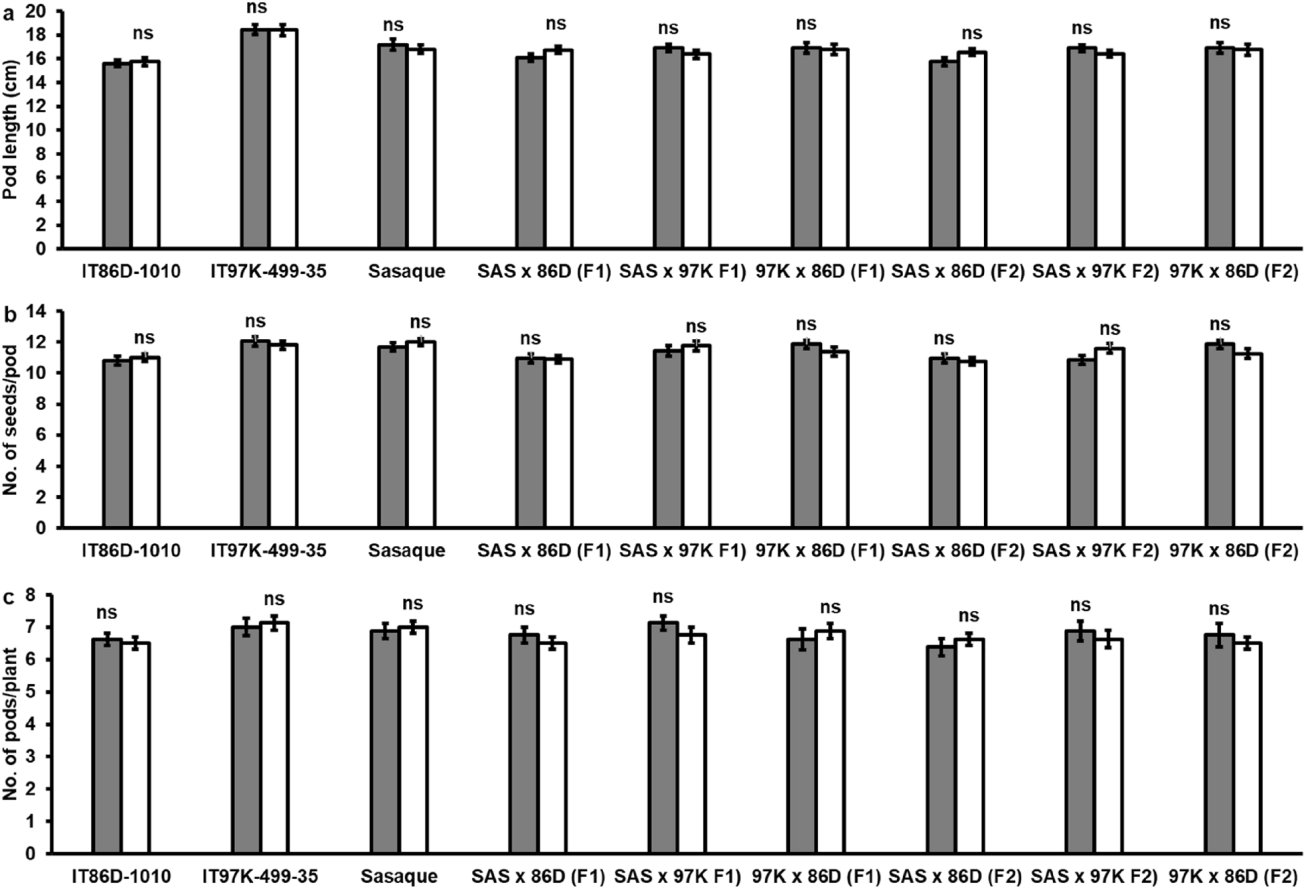
Comparison of **a** pod length, **b** number of seeds per pod and **c** number of pods per plant of plants cultivated from seeds allowed to dry on plants and plants cultivated from seeds of 14-day-old F_1_ pods oven-dried at 25 °C for 4 days, and plants cultivated from seeds of 11-day-old F_2_ pods oven-dried at 39 °C for 2 days

We compared the development of plants from seeds of oven-dried immature pods with those cultivated from seeds allowed to dry in situ on the plants. As satisfactory emergence and growth of seedlings were observed only in the plants cultivated with the seeds of 14-day-old pods oven-dried at 25 °C for 4 days and 11-day-old pods oven-dried at 39 °C for 2 days, plants from these two treatments were transplanted for further observation. In the F_1_ and F_2_ generations, days to flowering, pod length, number of seeds/pod and number of pods/plant of the plants in the control did not differ significantly from those of the two treatments (Table 3, Fig. 4). The difference in heights of seedlings between the control plants and plants of the seeds of 11-day-old pods oven-dried at 39 °C without silica gel (Table 2) had no significant effect on days to flowering (Table 3). These data confirm that cultivating seeds of 11-day-old oven-dried immature pods, which potentially results in 7 to  8 generations per year, has no significant effect on plant development, reproduction and pod yield components.

### Validation of the hybridity of the F_1_ plants

**Fig. 5:**
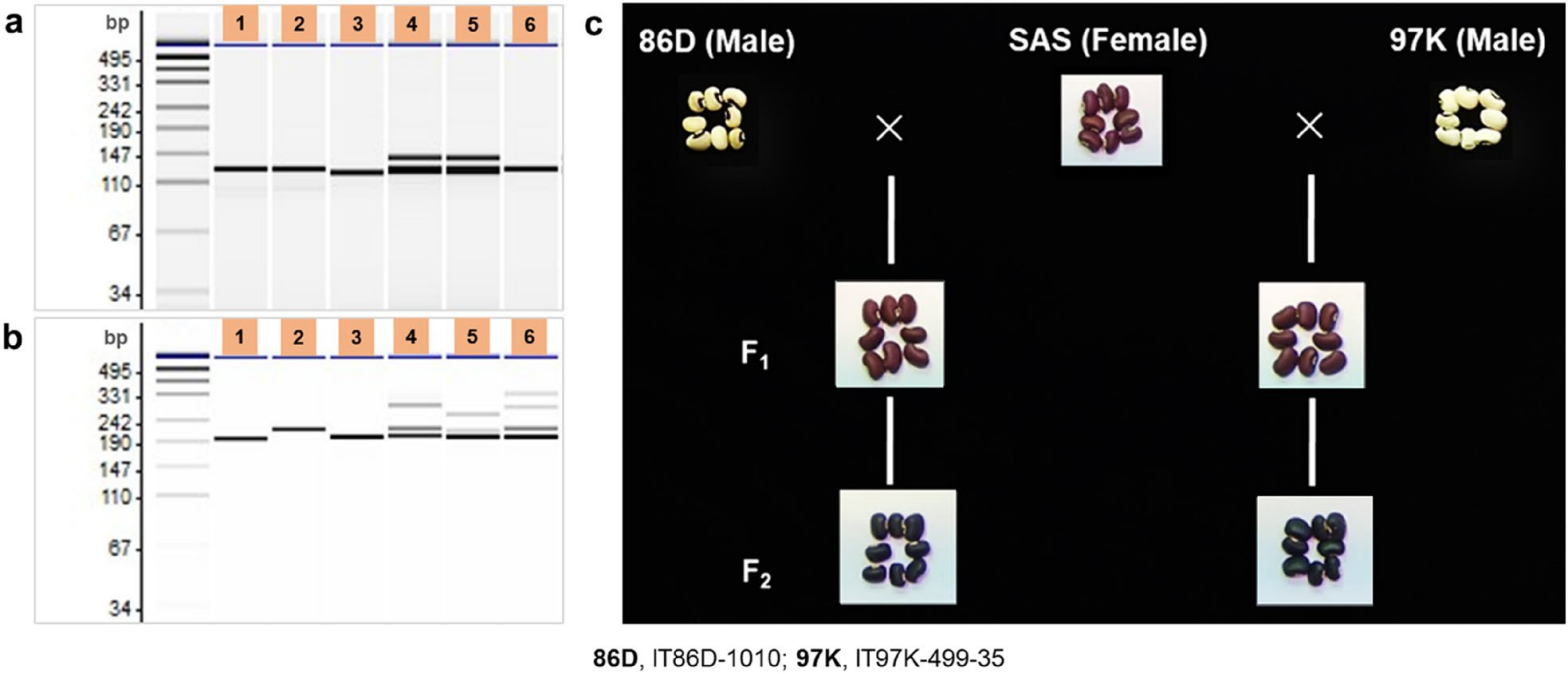
Confirmation of the hybridity of F_1_ hybrids. **a** Polymorphic SSR marker amplifications confirming the hybridity of Sasaque × IT86D-1010 F_1_ and Sasaque × IT97K-499-35 F_1_ hybrids. **b** polymorphic SSR marker amplifications confirming the hybridity of IT97K-499-35 × IT86D-1010 F_1_ hybrid. **c.** Black seed coat color of F_2_ seeds confirming the hybridity of Sasaque × IT86D-1010 and Sasaque × IT97K-499-35 F_1_ plants. Seed coat colors of F_1_ and F_2_ seeds are brown and black, respectively

Polymorphic amplifications of SSR markers in the parents and the corresponding F_1_ hybrid groups confirmed the hybridity of the F_1_ generation before the plants were cultivated for further observation. The parental genotypes were differentiated from each other, and the detection of double bands, indicating heterozygosity, differentiated the F_1_ hybrids from the corresponding parents (Fig. 5a, b). The black seed coat color of the F_2_ seeds of Sasaque × IT86D-1010 and Sasaque × IT97K-499-35 crosses (Fig. 5c), also provided a typical phenotypic marker of heterozygosity in the F_1_ generation. Cowpea crosses involving genotypes with white and brown seed coat colors produce F_2_ seeds with black seed coat color [33, 34].

## Discussion

Increasing the number of breeding generations per year is highly advantageous in breeding programs for any crop, particularly in species with long generation time. For a crop with a short generation time, the environmental requirements of the species may only be naturally met during a specific period of the year, outside of which the crop cannot be cultivated in the field. This confines the cultivation of many economically important crops, including cowpea, to a particular favorable season that occurs once per year. The integration of molecular breeding techniques into plant breeding programs has helped to reduce the number of breeding generations needed to develop an improved cultivar expressing a trait(s) of interest; however, it is impossible to completely avoid genotyping and phenotyping breeding populations over generations to fix genes and confirm stable transmission of the loci of interest. Although important advances in cowpea research have been made [6, 9, 10, 12, 14, 35–41], cowpea research outputs can hardly be compared to those of other important crops, despite cowpea's potential role in global food security [42, 43]. To fast-track development of improved cultivars, speed breeding protocols have been developed for major crops, including wheat [44, 45], rice [26, 27, 46], peanut [47] and soybean [25, 27], but the current study is the first such protocol for cowpea.

We found that neither days to flowering nor maturity was changed by elevated [CO_2_] (Table 2). A similar result was reported for soybean, another important legume crop, where it was concluded that flowering time of soybean in growth chambers is regulated by light and temperature, but not [CO_2_] [25]. Although CO_2_ supplementation had no effect on cowpea flowering time, the observed varying sensitivity of the genotypes to CO_2_ supplementation or CO_2_ supplementation-induced reduction in g_sw_ is informative. The average number of pods per plant in IT97K-499-35 was significantly increased in response to elevated [CO_2_], but there was no significant difference in the average number of pods per plant in Sasaque and IT86D-1010 under supplemented CO_2_ growth condition (Fig. 1c). Exposure of C_3_ plants, including cowpea, to elevated [CO_2_] mainly results in increased net photosynthesis and reduced g_sw_, but over time, the increase in photosynthesis is often offset by downregulation of photosynthetic capacity; hence, the photosynthetic gain from high [CO_2_] is not as high as may be expected [48–50]. Therefore, we attribute the small increases in pod length and number of seeds per pod observed in the LED (+) chambers, although not statistically significant, to increased net photosynthesis. The significant increase in pods/plant in IT97K-499-35, irrespective of the 71% decrease in stomatal conductance (Fig. 1c, d), indicates that the genotype may benefit from reduced evapotranspiration, may be insensitive to reduced stomatal performance, or has better water use efficiency than do the other genotypes [48, 49, 51]. Germination, reproduction and pod yield components of plants cultivated from seeds of 11-day-old pods oven-dried at 39 °C for 2 days have been shown to be comparable to those of plants cultivated from seeds allowed to dry on plants before harvesting (Tables 2, 3, Fig. 4), an indication that the time to harvest immature pods for an accelerated generation advance in cowpea breeding is 11 DAP. Pod elongation in the 3 genotypes of cowpea ceased by 11 DAP, which indicates that physiological development of cowpea seed is likely to be close to its peak by 11 DAP. Although we did not measure other pod development indices, our observation on pod elongation of cowpea is consistent with a previous study which recorded maximum values for cowpea pod development parameters by 15 days after anthesis [32]. The inclusion of silica gel, a desiccant, in the oven-drying process reduces the moisture content of the immature seeds to a level close to the moisture content of seeds allowed to dry on plants, resulting in improved germination and vigorous seedling development.

## Conclusions

From the results obtained under the growth chamber conditions reported in this study, it is possible to achieve between 5 and 6 breeding generations per year for cowpea genotypes that flower between 32 and 44 DAS. With the integration of cultivation of seeds of 11-day-old pods oven-dried at 39 °C for 2 days, the potential breeding generations per year can be further raised to approximately 7 for IT86D-1010 and IT97K-499-35, and 8 for Sasaque. Using the speed breeding protocol we have developed, an 8-generation breeding program involving Sasaque as a background, for example, which would take 8 years to complete in the field, can be completed in one year. As this protocol has no special technical requirements, it can be implemented in any standard growth chamber. Noteworthy is that plants cultivated from oven-dried immature pods show no defect at any stage of development. The integration of this protocol with high-throughput phenotyping and molecular breeding techniques will accelerate development of improved cowpea cultivars.

## Materials and methods

### Plant materials and growth conditions

We studied three semi-erect cowpea genotypes, Sasaque, IT86D-1010 and IT97K-499-35, in growth chambers illuminated with either, white light-emitting diodes (LED) or metal halide lamps (Table 1). Sasaque is a Japanese cultivar with a dark brown seed coat color, whereas IT86D-1010 and IT97K-499–-35 are breeding lines developed by International Institute of Tropical Agriculture, Nigeria. The two Nigerian lines have a white seed coat color and have been previously characterized [13, 29].

The main differences between the two chamber types are their sizes, light source and light intensities. The growth conditions set for plant germination and growth followed the recommended optimum conditions for cowpea cultivation in growth chambers [30], whereas the conditions adopted for hand pollination were determined empirically from the rate of successful hand crosses in this study (Table 1).

### CO_2_ supplementation and hand pollination

In a preliminary trial, we observed the effect of CO_2_ supplementation on the timing of flowering and maturation in each of the 3 genotypes. Four plants of each genotype were grown in 2 LED chambers, 1 of which was supplied with 1000 ppm of CO_2_. Crosses were made, in preparation for a further evaluation in the main trial. In the parental generation of the main study, 4 plants of each genotype were maintained in 4 LED chambers, resulting in a total of 12 plants per chamber and 16 plants per genotype, completely randomized within a plant cultivation tray (56 cm × 40 cm × 10 cm) placed in each of the chambers. To evaluate the response of the cowpea genotypes to elevated [CO_2_], 2 of the 4 LED chambers were supplied with 1000 ppm CO_2_ during the 10 h photoperiod. Other environmental conditions were kept constant in the 4 LED chambers (Table 1). For each genotype, we recorded days to 50% flowering and maturity, pod length, number of seeds per pod, number of pods per plant and stomatal conductance. To compare cross pollination success rates under other growth conditions, another 72 plants (24 plants per genotype) were raised in metal halide lamp chambers A and B (Table 1). Four plants per genotype were also completely randomized in 6 trays placed in the chambers, 3 trays per chamber. At the onset of flowering of Sasaque, the earliest flowering of the 3 genotypes, 2 trays were transferred to metal halide lamp chamber C (Table 1), in preparation for hand pollination. In the parental generation, only time to 50% flowering maturation of the plants in the metal halide lamp chambers were recorded. In all chambers, seeds were directly sown in 20 cm-high plastic pots filled with 1000 cm^3^ of granular culture soil (Nippi-Engei-Baido, Nihon Hiryo Co. Ltd., Tokyo, Japan). As none of the genotypes is completely erect, plants were clipped to stakes, and the tips of the growing stems were cut when they reached the roof of the LED chambers. In the metal halide lamp chambers, the tips of the stems were cut when the vines grew about 5 cm above their stakes. Cutting the tips induced the growth of branches, which were similarly managed to minimize entanglement between plants and ease hand pollination.

Crosses were made in one direction, resulting in 3 hybrid pairs: Sasaque × IT86D-1010, Sasaque × IT97K-499-35 and IT97K-499-35 × IT86D-1010. Four plants per parental genotype in the 5 experimental conditions: LED (−), LED (+), and metal halide lamps A, B and C (Table 1) were designated as female parents for cross pollinations, and 3 plants, 1 per genotype, constituted a replicate, resulting in 4 replicates per treatment. To maintain self-pollinated pods in the experimental plants for measurements of pod development (particularly in the LED chambers), pollen donors were sown one week ahead of the experimental plants in a different chamber under the same environmental conditions as those of the chamber designated as metal halide lamp A. All hand pollinations were completed within the first week of flower opening. Opened flowers were collected between 9:30 am and 10:00 am and preserved at 4 °C. At 6:00 pm that day, preserved flowers were removed from cold storage and kept at room temperature for 1 h to ensure pollen drying before attempting hand pollination. Between 7:00 pm and 8:00 pm, mature flower buds were emasculated and immediately pollinated with the preserved pollen grains. Flower buds on the same peduncle with the pollinated buds were nipped to eliminate competition for available nutrients. Crosses were deemed successful if pods formed from the pollinated pistils and the pods remained attached to the peduncle 3 days after pollination.

### Oven-drying of immature pods and germination tests

In the F_1_ generation, 14-day-old hybrid pods harvested from the parental generation were oven-dried at 25 °C for 4 days. A germination test was set up in metal halide chamber A to measure differences in emergence rates and heights of seedlings from seeds allowed to naturally dry on plants and seedlings from the 14-day-old oven-dried seeds. To further reduce the length of the reproductive phase of cowpea in our experiment, in the F_2_ generation, we evaluated emergence rates and growth of seedlings from seeds of 7- to 11-day-old pods oven-dried at 39 °C for 2 days at 25, 30, 35, 36, 37, 38, 39, 40, 41, 42, 43, 44 or 45 °C. More seeds of 11-day-old pods oven-dried at 39 °C for 1 day with or without the inclusion of silica gel in the drying oven, and 2 days with the inclusion of silica gel in the drying oven were also studied.

In each of the generations, 10 seeds in 3 replicates were sown, and emergence rates were evaluated 7 DAS, whereas heights of seedlings were measured 10 DAS. Only heights of seedlings that emerged by 7 DAS were measured. Seeds were sown in small plastic pots filled with 250 cm^3^ of Nipp-Engei-Baido soil, 1 seed per pot. Based on the performance of seedlings from the various treatments (see “Results” section), the moisture contents of more seeds of the parental genotypes from 11-day-old pods oven-dried at 39 °C for 2 days with or without the inclusion of silica gel, 14-day-old pods oven-dried at 25 °C for 4 days, and seeds allowed to naturally dry on plants were estimated using CD-6 Seed Moisture Content Meter (Shizuoka Seiki, Japan). To estimate the moisture contents of seeds under each treatment, 3 seeds were placed in the tray of the device in each measurement, and 3 measurements were taken for each treatment for the 3 cowpea genotypes (parents).

### Evaluation of time to flowering and maturation of plants cultivated from oven-dried seeds in F_1_ and F_2_ generations

In each of the two generations, 4 vibrant seedlings per genotype from the seedlings of 14-day-old seeds oven-dried at 25 °C for 4 days (F_1_) and 11-day-old pods oven-dried at 39 °C for 2 days were transplanted to large plastic pots, randomized in trays, and cultivated in metal halide chamber A. The pot size and volume of soil used were the same as the parental generation. We recorded days to 50% flowering and maturation, pod length, number of seeds per pod and number of pods per plant.

### Confirmation of the hybridity of F_1_ plants

Two pairs of polymorphic SSR markers (E354010801 and G6203) developed for cowpea genetic diversity analysis [8] were used to confirm the hybridity of the F_1_ plants. The markers were first confirmed to be polymorphic between each pair of parents, before being used to genotype the offspring of crosses. Genomic DNA samples from fresh young leaves of the parents and hybrid plants were isolated and purified using NucleoMag Plant DNA extraction kit (NucleoMag, Germany). A NanoDrop 2000 Ultraviolet–Visible Spectrophotometer (Thermo Fisher Scientific) was used to quantify and assess the quality of the extracted DNA samples. For each genotyping reaction, a 20 µL reaction volume containing 40 ng of genomic DNA, 1× KAPA Taq ReadyMix DNA Polymerase (KAPABiosystems) and 0.5 pmol/µL of forward and reverse primers was subjected to polymerase chain reaction (PCR) in a Bio-Rad thermal cycler (BIORAD, Japan). The recommended thermal cycling program for the markers [8] was adopted to amplify the SSR loci and the resulting PCR products were analyzed using a Microchip Electrophoresis System for DNA/RNA analysis (MultiNA, Shimadzu, Japan).

### Data recording and analysis

In the parental generation of the main study, stomatal conductance (g_sw_) was measured 25 days after sowing (DAS) using an LI-600 Porometer/Fluorometer (LI-COR Biosciences, USA). The manufacturer’s recommended instructions were followed for all measurements. With the Auto Mode of the device activated, a light-adapted measurement of g_sw_ of a single leaf on each experimental plant was taken, resulting in 16 measurements per genotype, 8 each in LED (−) and LED (+). Days to 50% flowering was recorded as the number of days from sowing to the time at least 50% of the plants flowered, and days to 50% maturity was measured as the number of days from sowing to the time at least 50% of the pods on each plant dried, since  flowering in cowpea is acropetal and the genotypes studied exhibit moderately indeterminate growth habit. To estimate the duration of pod elongation, all flowers (except for buds designated for cross pollination) were tagged on the day they opened, and 7 days after tagging, 12 fast-growing pods per genotype from the tagged flowers were selected for measurements, 6 each in LED (−) and LED (+). Using a string and a 50 cm rule, the length of each of the tagged pods was measured at 7, 11, and 14 DAP. In each genotype and under the two conditions [LED (−) and LED (+)], the numbers of seeds in the 12 pods selected for measurement of pod length were used to determine the average number of seeds per pod. Number of pods per plant was determined after the pods were harvested from all the experimental plants. To evaluate the effect of CO_2_ supplementation on g_sw_, pod development, and pod yield per plant, two-tailed t-tests were applied to compare the mean values of g_sw_ and the three pod yield components measured under LED (−) and LED (+).

In the F_1_ and F_2_ generations, we counted the number of seedlings that emerged from soil by 7 DAS, with which emergence rates were estimated under the various experimental conditions. Heights of seedlings were measured 10 DAS with the aid of a 50 cm rule under each of the study conditions. After transplanting of the desired seedlings, time to 50% flowering and maturity were measured for the plants cultivated from 14-day-old seeds oven-dried at 25 °C for 4 days (F_1_) and seeds of 11-day-old pods oven-dried for 2 days (F_2_). In each generation, two-tailed t-tests were applied to compare the mean values of the measured parameters under the control and oven-drying treatments. Microsoft Excel was used for t-tests.

## Supplementary Information

**Additional file 1: Table S1.** Raw data of hand pollination success rates under different growth chamber conditions (36 crosses per treatment).

## Data Availability

All data generated in this study, on which basis conclusions are made, are included in this published article. All materials are available through the corresponding author.

## References

[1] Oliveira M, Castro C, Coutinho J, Trindade H (2019). N supply and pre-cropping benefits to triticale from three legumes in rainfed and irrigated Mediterranean crop rotations. Field Crop Res.

[2] Sánchez-Navarro V, Zornoza R, Faz A, Fernández JA (2019). Does the use of cowpea in rotation with a vegetable crop improve soil quality and crop yield and quality? A field study in SE Spain. Eur J Agron.

[3] Takim FO (2012). Advantages of maize-cowpea intercropping over sole cropping through competition indices. J Agric Biodivers Res.

[4] FAOSTAT. Crops and livestock products: Food and Agriculture Organization of the United Nations. 2019. http://www.fao.org/faostat/en/#data/QCL/visualize. Accessed 23 Sep 2021

[5] Conrow J. Nigeria clears Bt cowpea for farmers’ use: alliance for science. 2019. https://allianceforscience.cornell.edu/blog/2019/12/nigeria-clears-bt-cowpea-for-farmers-use/. Accessed 5 Jan 2022

[6] Singh BB, IITA (2014). Cowpea: the food legume of the 21st century.

[7] Munoz-Amatriain M, Mirebrahim H, Xu P, Wanamaker SI, Luo M, Alhakami H (2017). Genome resources for climate-resilient cowpea, an essential crop for food security. Plant J.

[8] Chen H, Chen H, Hu L, Wang L, Wang S, Wang ML (2017). Genetic diversity and a population structure analysis of accessions in the Chinese cowpea [*Vigna unguiculata* (L.) Walp.] germplasm collection. Crop J.

[9] Boukar O, Belko N, Chamarthi S, Togola A, Batieno J, Owusu E (2019). Cowpea (*Vigna unguiculata*): genetics, genomics and breeding. Plant Breed.

[10] Boukar O, Fatokun CA, Huynh BL, Roberts PA, Close TJ (2016). Genomic tools in cowpea breeding programs: status and perspectives. Front Plant Sci.

[11] Bohra A, Pandey MK, Jha UC, Singh B, Singh IP, Datta D (2014). Genomics-assisted breeding in four major pulse crops of developing countries: present status and prospects. Theor Appl Genet.

[12] Che P, Chang S, Simon MK, Zhang Z, Shaharyar A, Ourada J (2021). Developing a rapid and highly efficient cowpea regeneration, transformation and genome editing system using embryonic axis explants. Plant J.

[13] Spriggs A, Henderson ST, Hand ML, Johnson SD, Taylor JM, Koltunow A (2018). Assembled genomic and tissue-specific transcriptomic data resources for two genetically distinct lines of Cowpea (*Vigna unguiculata* (L.) Walp.). Gates Open Res..

[14] Lonardi S, Munoz-Amatriain M, Liang Q, Shu S, Wanamaker SI, Lo S (2019). The genome of cowpea (*Vigna unguiculata* [L.] Walp.). Plant J.

[15] Ishii T, Juranic M, Maheshwari S, Bustamante FO, Vogt M, Salinas-Gamboa R (2020). Unequal contribution of two paralogous CENH3 variants in cowpea centromere function. Commun Biol.

[16] Ravi M, Chan SW (2010). Haploid plants produced by centromere-mediated genome elimination. Nature.

[17] Ishii T, Karimi-Ashtiyani R, Houben A (2016). Haploidization via chromosome elimination: means and mechanisms. Ann Rev Plant Biol.

[18] Karimi-Ashtiyani R, Ishii T, Niessen M, Stein N, Heckmann S, Gurushidze M (2015). Point mutation impairs centromeric CENH3 loading and induces haploid plants. Proc Natl Acad Sci USA.

[19] Ghosh S, Watson A, Gonzalez-Navarro OE, Ramirez-Gonzalez RH, Yanes L, Mendoza-Suarez M (2018). Speed breeding in growth chambers and glasshouses for crop breeding and model plant research. Nat Protoc.

[20] Watson A, Ghosh S, Williams MJ, Cuddy WS, Simmonds J, Rey M-D (2018). Speed breeding is a powerful tool to accelerate crop research and breeding. Nat Plants.

[21] Hickey LT, Amber NH, Robinson H, Jackson SA, Leal-Bertioli SCM, Tester M (2019). Breeding crops to feed 10 billion. Nat Biotechnol.

[22] Singh H, Janeja HS (2021). Review: speed breeding a ray of hope for the future generation in terms of food security. Eur J Mol Clin Med.

[23] Saxena KB, Saxena RK, Hickey LT, Varshney RK (2019). Can a speed breeding approach accelerate genetic gain in pigeonpea?. Euphytica.

[24] Samineni S, Sena M, Sajja SB, Gaur PM (2019). Rapid generation advance (RGA) in chickpea toproduce up to seven generations per year andenable speed breeding. Crop J.

[25] Nagatoshi Y, Fujita Y (2019). Accelerating soybean breeding in a CO_2_-supplemented growth chamber. Plant Cell Physiol.

[26] Tanaka J, Hayashi T, Iwata H (2016). A practical, rapid generation-advancement system for rice breeding using simplified biotron breeding system. Breeding Sci.

[27] Jahne F, Hahn V, Wurschum T, Leiser WL (2020). Speed breeding short-day crops by LED-controlled light schemes. Theor Appl Genet.

[28] Nuhu Y, Mukhtar FB (2013). Screening of some cowpea genotypes for photosensitivity. Bayero J Pure Appl Sci.

[29] Salinas-Gamboa R, Johnson SD, Sanchez-Leon N, Koltunow AM, Vielle-Calzada JP (2016). New observations on gametogenic development and reproductive experimental tools to support seed yield improvement in cowpea [*Vigna unguiculata* (L.) Walp]. Plant Reprod.

[30] Angelotti F, Barbosa LG, Barros JRA, Santos CAF (2020). Cowpea development under different temperatures and carbon dioxide concentrations. Pesqui Agropecu Trop.

[31] Cavalcante EG, de Medeiros JF, Sobrinho JE, Figueiredo VB, da Costa JPN, Santos WD (2016). Development and water requirements of cowpea under climate change conditions in the Brazilian semi-arid region. Rev Bras Eng Agr Amb.

[32] Cruz JMFL, Alves EU, Farias OR, Araújo PC, Oliveira AP (2019). Physiological maturity and determination of the harvest time of *Vigna unguiculata* L. Walp. JEAI.

[33] Ajayi AT, Gbadamosi AE, Olotuah OF, David EA (2020). Crossability and inheritance of seed coat colour in cowpea at F1 generation. Front Life Sci RT.

[34] Egbadzor KF, Yeboah M, Gamedoagbao DK, Offei SK, Danquah EY, Ofori K (2014). Inheritance of seed coat colour in cowpea (*Vigna unguiculata* (L.) Walp). Int J Plant Breed Genet.

[35] Paudel D, Dareus R, Rosenwald J, Munoz-Amatriain M, Rios EF (2021). Genome-wide association study reveals candidate genes for flowering time in cowpea (*Vigna unguiculata* [L.] Walp.). Front Genet.

[36] Zuluaga DL, Lioi L, Delvento C, Pavan S, Sonnante G (2021). Genotyping-by-sequencing in *Vigna unguiculata* landraces and its utility for assessing taxonomic relationships. Plants.

[37] Hall AE (2003). Future directions of the bean/cowpea collaborative research support program. Field Crop Res.

[38] Lo S, Munoz-Amatriain M, Hokin SA, Cisse N, Roberts PA, Farmer AD (2019). A genome-wide association and meta-analysis reveal regions associated with seed size in cowpea [*Vigna unguiculata* (L.) Walp]. Theor Appl Genet.

[39] Das S, Bhat PR, Sudhakar C, Ehlers JD, Wanamaker S, Roberts PA (2008). Detection and validation of single feature polymorphisms in cowpea (*Vigna unguiculata* L. Walp) using a soybean genome array. BMC Genomics.

[40] Das S, Ehlers JD, Close TJ, Roberts PA (2010). Transcriptional profiling of root-knot nematode induced feeding sites in cowpea (*Vigna unguiculata* L. Walp.) using a soybean genome array. BMC Genomics.

[41] Juranic M, Nagahatenna DSK, Salinas-Gamboa R, Hand ML, Sanchez-Leon N, Leong WH (2020). A detached leaf assay for testing transient gene expression and gene editing in cowpea (*Vigna unguiculata* [L.] Walp.). Plant Methods.

[42] Silva ACD, Santos DDC, Junior DLT, Silva PBD, Santos RCD, Siviero A. Cowpea: a strategic legume species for food security and health. 2019. In: legume seed nutraceutical research. IntechOpen; [86]. https://www.intechopen.com/chapters/62227. Accessed 10 Aug 2021

[43] Gomes AMF, Nhantumbo N, Ferreira-Pinto M, Massinga R, Ramalho JC, Ribeiro-Barros A, El-Esawi MA (2019). Breeding elite cowpea [*Vigna unguiculata* (L.) Walp] varieties for improved food security and income in Africa: opportunities and challenges. Legume crops: characterization and breeding for food security.

[44] Alahmad S, Dinglasan E, Leung KM, Riaz A, Derbal N, Voss-Fels KP (2018). Speed breeding for multiple quantitative traits in durum wheat. Plant Methods.

[45] Christopher J, Richard C, Chenu K, Cristopher M, Borrell A, Hickey L (2015). Integrating rapid phenotyping and speed breeding to improve stay-green and root adaptation of wheat in changing, water-limited Australian environments. Proc Environ Sci.

[46] Ohnishi T, Yoshino M, Yamakawa H, Kinoshita T (2011). The biotron breeding system: a rapid and reliable procedure for genetic studies and breeding in rice. Plant Cell Physiol.

[47] O'Connor DJ, Wright GC, Dieters MJ, George DL, Hunter MN, Tatnell JR (2013). Development and application of speed breeding technologies in a commercial peanut breeding program. Peanut Sci.

[48] Long SP, Ainsworth EA, Rogers A, Ort DR (2004). Rising atmospheric carbon dioxide: plants FACE the future. Annu Rev Plant Biol.

[49] Leakey AD, Ainsworth EA, Bernacchi CJ, Rogers A, Long SP, Ort DR (2009). Elevated CO2 effects on plant carbon, nitrogen, and water relations: six important lessons from FACE. J Exp Bot.

[50] Bernacchi CJ, Leakey AD, Heady LE, Morgan PB, Dohleman FG, McGrath JM (2006). Hourly and seasonal variation in photosynthesis and stomatal conductance of soybean grown at future CO(2) and ozone concentrations for 3 years under fully open-air field conditions. Plant Cell Environ.

[51] Long SP, Ort DR (2010). More than taking the heat: crops and global change. Curr Opin Plant Biol.

