# Supplementary material for: Cowpea speed breeding using regulated growth chamber conditions and seeds of oven-dried immature pods potentially accommodates eight generations per year
Source: Plant Methods. 2022 Aug 29;18:106. doi: 10.1186/s13007-022-00938-3 (PMC9422124; doi:10.1186/s13007-022-00938-3)
Supplement: Supplementary file 1 — Additional file 1: Table S1. Raw data of hand pollination success rates under different growth chamber conditions (36 crosses per treatment). [file 13007_2022_938_MOESM1_ESM.docx]

**Table S1** Raw data of hand pollination success rates under different growth chamber conditions (36 crosses per treatment)

| Growth chamber | Success rates of replicated hand pollinations (%) | | | |
| --- | --- | --- | --- | --- |
|  | Rep 1 | Rep 2 | Rep 3 | Rep 4 |
| LED (-) | 70 | 60 | 80 | 70 |
| LED (+) | 60 | 70 | 70 | 60 |
| MHL A | 10 | 30 | 10 | 10 |
| MHL B | 0 | 10 | 0 | 10 |
| MHL C | 50 | 40 | 60 | 50 |

LED, light-emitting diode; MHL, metal halide lamp; A – C, growth conditions described in Table 1
